# Cognitive Frailty and Its Risk Factors Among Patients With Chronic Kidney Disease Receiving Hemodialysis: A Cross‐Sectional Study

**DOI:** 10.1111/nhs.70197

**Published:** 2025-07-23

**Authors:** Mu‐Hsing Ho, Pei‐Chi Chen, Megan F. Liu, Jung Jae Lee

**Affiliations:** ^1^ School of Nursing, LKS Faculty of Medicine The University Hong Kong Pokfulam Hong Kong; ^2^ Hemodialysis Center Cardinal‐Tien Hospital New Taipei City Taiwan; ^3^ School of Gerontology and Long‐Term Care Taipei Medical University Taipei Taiwan

**Keywords:** chronic kidney disease, cognitive frailty, depressive symptoms, hemodialysis, nutrition

## Abstract

Cognitive frailty can lead to an impaired functional capacity and a poor quality of life, especially in patients on hemodialysis. This study aimed to investigate cognitive frailty and its risk factors in patients with chronic kidney disease (CKD) receiving hemodialysis. A cross‐sectional study was conducted between April and June 2021 involving 220 patients with CKD receiving hemodialysis at a hospital‐based hemodialysis center in northern Taiwan. Data were collected using a structured survey covering demographics, cognitive function, physical frailty, depressive symptoms, physical activity, and nutritional status. Univariate and multivariate logistic regression models were used to identify risk factors for cognitive frailty. In total, 220 patients were recruited. Prevalences of cognitive impairment, physical frailty, depressive symptoms, and cognitive frailty were 46.8%, 10.0%, 52.3%, and 9%, respectively. Univariate and multivariate logistic regression analyses, adjusted for age, sex, and years of hemodialysis, identified malnutrition (aOR = 12.405; 95% CI = 3.29–46.81) and physical inactivity (aOR = 89.445; 95% CI = 5.87–1363.93) as significant risk factors for cognitive frailty. The study suggests the need for strategies to enhance physical activity and nutritional status to prevent cognitive frailty in patients with CKD receiving hemodialysis.

**Summary**

- While cognitive frailty is clinically relevant to chronic kidney disease (CKD), cognitive frailty and its associated risk factors among patients with CKD undergoing hemodialysis are understudied.
- Malnutrition and physical inactivity in patients with CKD are significant risk factors for cognitive frailty.
- Healthcare professionals including nurses should prioritize nursing interventions that improve nutritional status and encourage physical activity in hemodialysis patients with CKD to reduce cognitive frailty.

## 1 Introduction

Globally, more than 10% of the world's population is suffering from chronic kidney disease (CKD) (Kovesdy 2022). CKD is a rapidly growing health burden and is projected to become the fifth leading cause of lost years of life by 2040 (Foreman et al. 2018). In particular, patients with CKD often experience “premature ageing” symptoms such as osteoporosis, muscle wasting, cardiovascular hypertrophy, and vascular calcification (Kooman et al. 2014). This phenomenon resembles common systemic complications in patients with other chronic diseases such as human immunodeficiency virus infection, chronic obstructive pulmonary disease, and rheumatoid arthritis (Yegorov et al. 2020). The physiological mechanisms underlying CKD, such as endothelial dysfunction, atherosclerosis, a chronic inflammatory state, and activation of oxidative stress functions, put patients at high risk of experiencing the above co‐occurring morbidities as well as physical and psychological symptom burdens such as fatigue, sleep disturbances, cognitive function declines, and depressive symptoms (Nixon et al. 2018; Zheng et al. 2022). Managing CKD requires a comprehensive assessment of both blood test results and patient‐reported outcomes, as well as active engagement of patients with CKD in lifestyle modifications (Chen et al. 2019). Poor management of CKD can lead to early experience of the aforementioned burdens such as frailty, cognitive impairment, and depressive symptoms (McAdams‐DeMarco et al. 2013; Nascimento et al. 2014).

### 1.1 Frailty in Patients With CKD

Frailty is a condition in which multiple physiological systems have progressively deteriorated, thereby increasing one's susceptibility to physical stressors, and is prevalent in patients with CKD. Those on hemodialysis are the most frail (Chowdhury et al. 2017; Clegg et al. 2013). A meta‐analysis showed that 46% of patients with end‐stage renal disease on hemodialysis have subjective feelings of being frail (Lee and Son 2021). Frailty might not always accompany aging; the overall prevalence of frailty in healthy older adults living in the community was around 10% (Collard et al. 2012; He et al. 2019). However, older people with CKD can be more fragile than healthy older people when health management is inadequately performed because they experience both aging‐ and renal disease‐related impairments (Nitta et al. 2017). A study conducted among patients on hemodialysis in the United States (*n* = 1576) found that frailty was significantly correlated with mortality after controlling for the estimated glomerular filtration rate (Bao et al. 2012). Moreover, the European Renal Best Practice Group also recommends that screening for frailty should be added to the standard practice of managing older patients with CKD, and those patients at high risk of developing frailty should receive tailor‐made interventions to prevent or alleviate the frailty‐related implications as CKD is an independent predictor for frailty (Farrington et al. 2017). It is particularly crucial to identify patients in a pre‐frail condition (i.e., at high risk of developing frailty) by implementing frailty preventive initiatives.

### 1.2 Cognitive Frailty in Patients With CKD

According to a concept from the international consensus group of the International Academy on Nutrition and Aging (IANA) and the International Association of Gerontology and Geriatrics (IAGG), cognitive frailty refers to physical frailty and cognitive impairment. As cognitive impairment is highly associated with neuropsychiatric symptoms, particularly in depression, it was suggested to add a brief psychological assessment (e.g., depressive symptoms assessment) together with a cognitive and physical frailty assessment when screening for cognitive frailty (Kelaiditi et al. 2013). Recently, research has integrated cognition in the definition of frailty instead of only focusing on the physical domain (Chang et al. 2022). Buchman and colleagues found that the development of physical frailty was independently correlated with an accumulation of common brain pathological findings, such as Alzheimer's disease pathology, macroinfarcts, and nigral neuronal loss (Buchman et al. 2013). Frailty is a multidimensional syndrome characterized by increased vulnerability to stressors as a result of reduced capacity of different physiological systems. Cognitive impairment and its coexisting neuropsychiatric symptoms, particularly depression, are linked to physical frailty. However, these concepts (physical frailty, cognitive impairment, and depression) have traditionally been studied separately (Kelaiditi et al. 2013).

Cognitive frailty is clinically relevant to CKD because the accumulated deficits of frailty, cognitive functional declines, and depressive symptoms can lead to an impaired functional capacity and a poor quality of life, especially in patients on hemodialysis (Chowdhury et al. 2017; Kelaiditi et al. 2013). In patients with CKD, depression independently increases cognitive frailty risk (OR = 2.52) and interacts with low social support to accelerate decline. Furthermore, comorbid conditions such as the presence of diabetes, hypertension, and cardiovascular disease further increase the risk of cognitive frailty in patients with CKD (Chang et al. 2022). Their capacity to carry out activities of daily living may be hampered by physical restrictions brought on by frailty and limitations associated with receiving hemodialysis, that is, weakness in their arteriovenous fistula arm. Evidence from a meta‐analysis showed that in community‐dwelling older people, those in a frail state experienced a significantly lower quality of life in both mental and physical domains than those in a more robust condition (Kojima et al. 2016). Early screening and evaluation of cognitive frailty have the potential to produce significant benefits for patients with CKD. In addition, timely care of cognitive frailty deficiencies is essential to identifying predisposing factors and limiting the propagation of deterioration in the quality of life (Chowdhury et al. 2017; Greinert et al. 2022). To our knowledge, cognitive frailty and its associated risk factors among patients with CKD undergoing hemodialysis are understudied. Therefore, we aimed to assess the prevalence of cognitive frailty and risk factors for cognitive frailty among patients with CKD receiving hemodialysis. There is no standardized measure for assessing cognitive frailty in patients with CKD receiving hemodialysis. The operational definition of cognitive frailty was co‐existing conditions of cognitive functioning decline, physical frailty, and depressive symptoms. This study investigated and reported on the prevalence of cognitive frailty and its risk factors.

## 2 Methods

### 2.1 Study Design and Samples

A descriptive, cross‐sectional study was undertaken in April to June 2021 in a hospital‐based hemodialysis center located in northern Taiwan. Patients were enlisted through convenience sampling. The inclusion criteria were (1) aged 18 years and older; (2) currently receiving regular hemodialysis, at least once a week; and (3) able to communicate in Mandarin Chinese. Patients with CKD who had received hemodialysis for less than 3 months, had been admitted to a hospital during the survey period, or had a single‐pool volume cleared/volume distribution (*Kt*/*V*) value of < 1.0 were excluded. Additionally, we excluded patients with CKD having concurrent Alzheimer's disease and other types of dementia. We conducted a sample size estimation using G* power vers. 3.0.10 software (Faul et al. 2007). The statistical test and model settings for the sample size estimation were as follows: *t* tests as the test family; and means: difference between two independent means (given *α*, power, and effect size). Parameter settings (*α* = 0.05, 1 – *β* = 0.90, and effect size *d* = 0.50) were established according to a previous study on self‐reported health changes in hemodialysis patients modulating the effect of frailty (Anderson et al. 2022), and a total sample of 140 was suggested. An attrition rate of 30% was considered, and thus an estimated sample size of 200 participants was determined and considered sufficient. Strengthening the Reporting of Observational Studies in Epidemiology (STROBE) reporting guidelines for cross‐sectional studies were followed (von Elm et al. 2008).

### 2.2 Data Collection

Between April and June 2021, we conducted a structured survey at a hospital‐based hemodialysis center in northern Taiwan. The center has 61 beds for hemodialysis and can accommodate up to 183 patients per day. A research assistant invited potential participants either before or after their hemodialysis sessions. To minimize selection bias, we ensured that invitations were extended to a diverse range of patients, including those at different stages of CKD, with varying durations of hemodialysis treatment, and across different demographic groups. Additionally, we used random sampling to select participants from the eligible pool, aiming to achieve a representative sample.

The survey consisted of (1) demographics, (2) cognitive function, (3) physical frailty, (4) depressive symptoms, (5) physical activity, and (6) nutritional status to obtain data. The research aim and ethics considerations were fully explained to patients by a trained research assistant before the commencement of data collection. Written informed consent was obtained from each eligible patient.

#### 2.2.1 Demographics

Demographic data including age, sex (female/male), educational level (elementary school, junior or senior high school, college/university and above), marital status (single/married), employment status (no/yes), residential status (living alone/living with others), years receiving hemodialysis, comorbidities, kidney transplant (no/yes), and smoking history (no/yes) were collected.

#### 2.2.2 Cognitive Function

Cognitive function was assessed by the Kidney Disease Quality of Life Cognitive Function (KDQOL‐CF) subscale items (Hays et al. 1997). Three items of the cognitive function subscale assess perceived attention and memory impairment by asking the patients, “Did you have difficulty concentrating or thinking?” “Did you react slowly to things that were said or done?” and “Did you become confused?” during the past 4 weeks. Each item is rated from 1 (none of the time) to 6 (all of the time). Items with a score of ≥ 2 were categorized as positive. The operational definition of cognitive impairment that referred to all three cognitive function subscale items was identified as positive. The KDQOL‐CF subscale is considered a valid screening instrument for assessing cognitive function in patients with CKD (Kurella et al. 2004). The traditional Chinese (Taiwan) version of the KDQOL‐CF was translated by FACITtrans and is available on RAND Health (http://www.rand.org/health/surveys_tools/kdqol.html). The KDQOL‐CF was previously used in the Chinese population (Chan et al. 2024).

#### 2.2.3 Physical Frailty

Frailty was evaluated using Fried's frailty phenotype scale (Fried et al. 2001). Fried and colleagues proposed a definition of frailty as a biological syndrome with a group of clinical signs and symptoms when an individual shows decreases in one or multiple phenotypic components (weight loss, exhaustion, physical activity, walking time, and grip strength). This definition was also used as an operational definition in this study. The component of weight loss refers to an unintentional weight loss of over 3 kg in the last year. Less physical activity was defined as < 383 kcal/week in men or < 270 kcal/week in women. Walking time refers to the time used to walk 15 ft (≈4.6 m). Individuals that met one or two of the criteria were considered to be in a pre‐frail condition, and those with more than two were in a frail condition. According to Fried's frailty criteria, the presence of one phenotypic component is scored 1 point, and the absence is scored zero. A total score of greater than 3 is considered “frailty.” The scale has been validated and widely used in the Taiwanese population (Tzeng et al. 2025; Wang et al. 2019).

#### 2.2.4 Depressive Symptoms

Depressive symptoms were assessed by the Center for Epidemiologic Studies Depression (CES‐D) scale. The CES‐D is a self‐reported, validated questionnaire, with 20 items measuring depressive symptomatology in the past week. Total scores range from 0 to 60, with higher scores indicating more severe depression and a cutoff score of 16 or more indicating clinically significant depressive symptoms (Radloff 1977). The CES‐D has been validated and tested in the Taiwanese population to measure depressive symptoms (Chin et al. 2015).

#### 2.2.5 Physical Activity

Physical activity was measured by the International Physical Activity Questionnaire‐Short Form (IPAQ‐SF) that includes seven items that ask about “the time you spent being physically active in the last 7 days” to respondents in terms of the time spent in high‐intensity and moderate‐intensity exercise, walking, or sitting (Lee et al. 2011). Outcomes of the IPAQ‐SF are in metabolic equivalents of task (METs)‐min/week. We categorized physical inactivity as < 600 METs‐ min/week based on recommendations of the World Health Organization (WHO) of at least 150 min/week of brisk walking or 75 min of running which is equivalent to 600 MET‐ min/week of physical activity. The IPAQ‐SF was validated in Chinese for the Taiwanese population (Liou et al. 2008).

#### 2.2.6 Nutritional Status

Nutritional status was assessed using the Short‐Form Mini Nutritional Assessment (MNA‐SF) (Rubenstein et al. 2001). The MNA‐SF is a validated nutritional assessment tool including six screening items. The total score ranged from 0 to 14 points, while a summary score of 11 or lower indicates a risk of malnutrition. The MNA‐SF was validated in Chinese and used for the Taiwanese population (Tsai et al. 2013).

### 2.3 Cognitive Frailty

The operational definition of cognitive frailty in this study was positive identification in all assessments of cognitive functioning decline (KDQOL‐CF), physical frailty (Fried's frailty phenotype), and depressive symptoms (CES‐D).

### 2.4 Data Analysis

All data were analyzed using IBM SPSS Statistics for Windows vers. 26.0 (IBM, Armonk, NY, USA). Descriptive analyses, including means and standard deviations (SDs) for continuous variables and frequencies and percentages for categorical variables, were used to summarize data distributions. Pearson's correlation and independent *t*‐tests were used to examine the interaction between physical frailty, cognitive functioning decline, and depressive symptoms. Chi‐squared test (Fisher's exact test was used if the expected value in any cell was less than 5) was used to test for differences between independent variables among patients receiving hemodialysis with and those without cognitive frailty. Univariate and multivariate logistic regression analyses were employed to determine the most critical risk factors associated with cognitive frailty among patients undergoing dialysis. For each outcome of interest, crude odds ratios (ORs), adjusted ORs (aORs), and corresponding 95% confidence intervals (CIs) were estimated. Statistical significance was set at a two‐tailed *p* value of < 0.05.

## 3 Results

We initially identified 227 participants, but 7 of them refused to complete the survey because they were unwilling to perform the grip strength test. Therefore, data from 220 patients receiving hemodialysis were utilized. Prevalences of cognitive impairment, physical frailty, depressive symptoms, and cognitive frailty were 46.8%, 10%, 52.3%, and 9%, respectively. Table 1 shows cognitive functioning declines, physical frailty, and depressive symptoms identified in this study. The mean age of patients was 45.3 (SD = 13.4) years with an average of 4.5 years of receiving hemodialysis. Among the study cohort, 56.8% (*n* = 125) were male, and 59.5% (*n* = 131) were married. Eighty‐five percent (*n* = 189) of patients were employed, and more than 90% (*n* = 206) were currently living with their family.

**TABLE 1:** Assessments for identifying cognitive frailty in patients (*N* = 220).

Assessment	Positive, *n*	Prevalence (%)
Cognitive functional decline^a^	103	46.8
Have difficulty concentrating or thinking?	153	69.5
React slowly to things that were said or done?	121	55.0
Become confused?	107	48.6
Physical frailty	22	10.0
Depressive symptoms	115	52.3
Cognitive frailty^b^	20	9.0

^a^ Question description: During the past 4 weeks, did you…?

^b^ Positively identified by cognitive functional declines, physical frailty, and depressive symptom assessments.

### 3.1 Relationships Between Physical Frailty, Cognitive Functioning, and Depressive Symptoms

**FIGURE 1:**
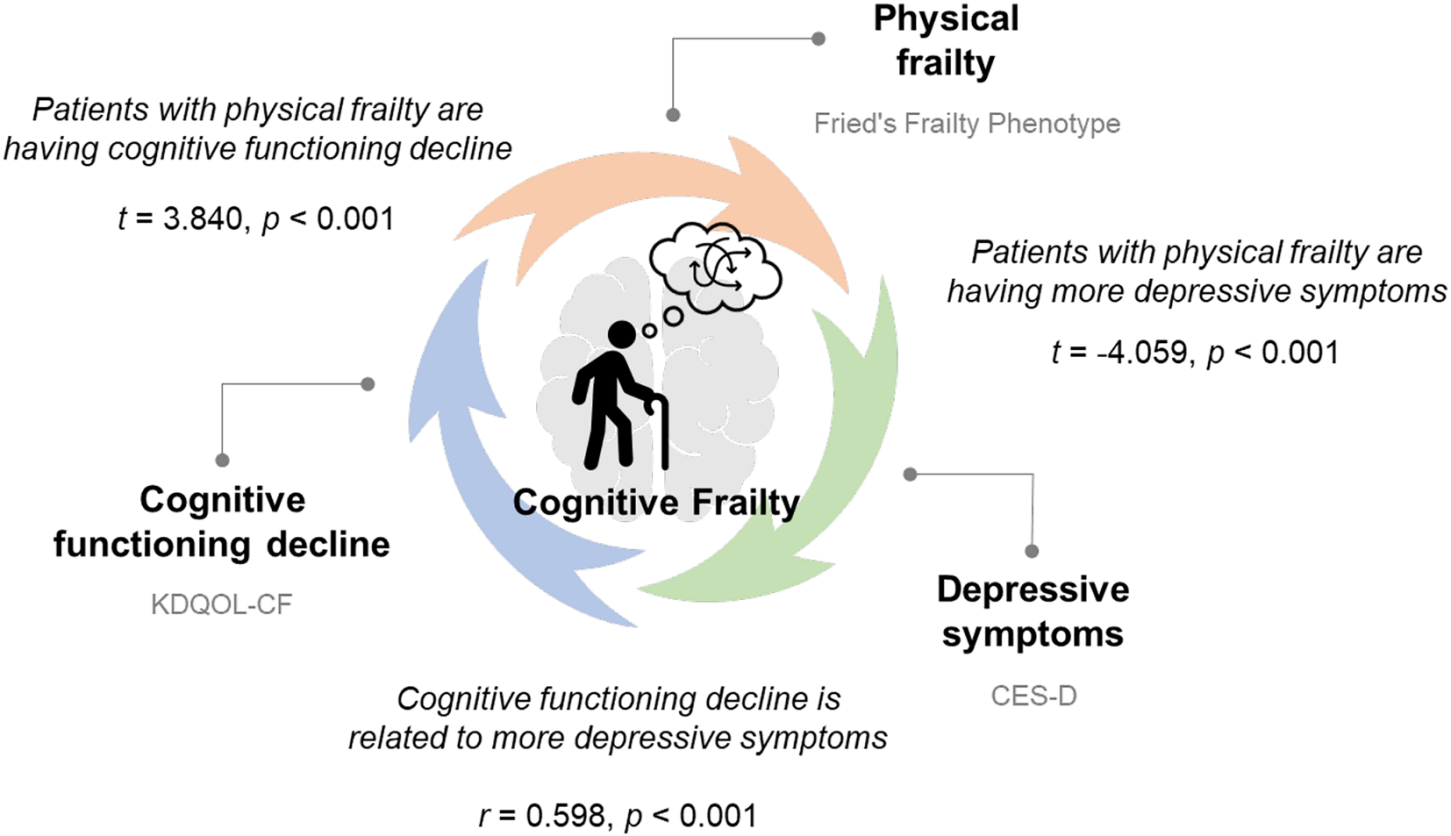
Relationships between physical frailty, cognitive functioning, and depressive symptoms. CES‐D, Center for Epidemiologic Studies Depression; KDQOL‐CF, Kidney Disease Quality of Life Cognitive Function.

Figure 1 demonstrates the relationships between the components of cognitive frailty, including physical frailty, cognitive functioning, and depressive symptoms. Patients with physical frailty had cognitive functioning decline (*t* = 3.840, *p* < 0.001) and more depressive symptoms (*t* = ‐4.059, *p* < 0.001) than patients without physical frailty. We also found that cognitive functioning decline was positively correlated to more depressive symptoms (*r* = 0.598, *p* < 0.001; moderately strong correlation).

### 3.2 Differences in Demographics Between Patients With and Those Without Cognitive Frailty

Table 2 presents comparisons of patients with and those without cognitive frailty. Patients characterized by cognitive frailty were on average 39.4 (SD = 12.7) years old, and those without cognitive frailty were aged 45.9 (SD = 13.3) years (*p* = 0.038). Significant differences in sex and smoking status related to cognitive frailty were observed, in that 70% of patients with cognitive frailty were female (*n* = 14), and 35% of patients with cognitive frailty smoked (*n* = 7). Statistical significance was also found between years of receiving hemodialysis and cognitive frailty, in that those patients with cognitive frailty (mean = 6.7, SD = 7.8) had longer average years of receiving hemodialysis than those without cognitive frailty (mean = 4.2, SD = 4.6; *p* = 0.037).

**TABLE 2:** Comparison of patients with or those without cognitive frailty (*N* = 220).

Variables		Cognitive frailty		
	Overall (*N* = 220)	Without (*N* = 200)	With (*N* = 20)	*p*
Age (years)	45.3 ± 13.4	45.9 ± 13.3	39.4 ± 12.7	**0.038**
18–40	77 (35.0)	65 (32.5)	12 (60.0)	
41–64	117 (53.2)	110 (55.0)	7 (35.0)	
65–87	26 (11.8)	25 (12.5)	1 (5.0)	
Sex, *n* (%)				
Female	95 (43.2)	81 (40.5)	14 (70.0)	**0.011**
Male	125 (56.8)	119 (59.5)	6 (30.0)	
Education level				0.506
Elementary school	8 (3.6)	8 (4.0)	0 (0.0)	
Junior or senior high school	122 (59.1)	112 (56.0)	10 (50.0)	
College/university and above	90 (40.9)	80 (40.0)	10 (50.0)	
Marital status, *n* (%)				
Single	89 (40.5)	80 (40.0)	9 (45.0)	0.664
Married	131 (59.5)	120 (60.0)	11 (55.0)	
Employment status, *n* (%)				
Unemployed	31 (14.1)	29 (14.5)	2 (10.0)	0.442^a^
Employed	189 (85.9)	171 (85.5)	18 (90.0)	
Residential status, *n* (%)				
Living alone	14 (6.4)	12 (6.0)	2 (10.0)	0.370^a^
Living with others	206 (93.6)	188 (94.0)	18 (90.0)	
Years receiving hemodialysis	4.5 ± 5.1	4.2 ± 4.6	6.7 ± 7.8	**0.037**
Comorbidities, yes				
Hypertension	157 (71.4)	143 (71.5)	14 (70.0)	0.887
Stroke	26 (11.8)	22 (11.0)	4 (20.0)	0.268^a^
Heart failure	6 (2.7)	4 (2.0)	2 (10.0)	0.094^a^
Diabetes mellitus	86 (39.1)	75 (37.5)	11 (55.0)	0.126
Dyslipidemia	7 (3.2)	5 (2.5)	2 (10.0)	0.125
Kidney transplant, yes	1 (0.5)	0 (0.0)	1 (5.0)	0.091
Smoking history, yes	29 (13.2)	22 (11.0)	7 (35.0)	**0.008** ^**a**^
Physical activity (METs)	1027.3 ± 1246.7	1104.2 ± 1281.6	258.3 ± 159.7	**< 0.001**
Insufficient (< 600 METs)	98 (45.5)	79 (39.5)	19 (95.0)	**< 0.001** ^a^
Sufficient (≥ 600 METs)	122 (55.5)	121 (60.5)	1 (5.0)	
Nutritional status	12.4 ± 1.8	12.7 ± 1.6	10.7 ± 2.2	**< 0.001**
Normal	167 (75.9)	159 (79.5)	8 (40.0)	**< 0.001** ^a^
At risk of malnutrition	53 (24.1)	41 (20.5)	12 (60.0)	

*Note:* Values in bold are statistically significant.

Abbreviation: METs, metabolic equivalents of task.

^a^ Fisher's exact test.

### 3.3 Differences in Physical Activity and the Nutritional Status Between Patients With and Those Without Cognitive Frailty

The mean level of physical activity in patients with cognitive frailty (mean = 258.3, SD = 159.7 METs‐min) was significantly lower than that of those without cognitive frailty (mean = 1104.2, SD = 1281.6 METs‐min; *p* < 0.001). Around 95% of patients with cognitive frailty (*n* = 19) were categorized as physically inactive (< 600 METs‐min), while over 60% of patients without cognitive frailty had sufficient physical activity (*p* < 0.001). As to the nutritional status, the mean score of the MNA‐SF in patients with cognitive frailty (mean = 10.7, SD = 2.2) was also significantly lower than that of patients without cognitive frailty (mean = 12.7, SD = 1.6; *p* < 0.001). Also, over 60% of patients with cognitive frailty were at risk of malnutrition (*p* < 0.001) (Table 2).

### 3.4 Factors Associated With Cognitive Frailty

The findings of the univariate and multivariate logistic regression analyses to determine factors associated with cognitive frailty are presented in Table 3. In the univariate logistic regression models, being at risk of malnutrition and reporting physical inactivity were two significant factors affecting cognitive frailty. After adjusting for patients' age, sex, and years of receiving hemodialysis, the multivariate logistic regression analysis revealed that being at risk of malnutrition (aOR = 12.405; 95% CI = 3.29–46.81) and being physically inactive (aOR = 89.445; 95% CI = 5.87–1363.93) were still significantly associated with an increased risk of cognitive frailty.

**TABLE 3:** Factors associated with cognitive frailty among patients undergoing hemodialysis (*N* = 220).

	Unadjusted univariate model			Adjusted multivariate model		
Variable	Crude OR	SE	95% CI	Adjusted OR	SE	95% CI
Age	0.957*	0.021	(0.92–0.99)	0.934**	0.026	(0.89–0.98)
Sex (reference: male)	3.428*	0.509	(1.27–9.29)	3.021	0.653	(0.84–10.54)
Years receiving hemodialysis	1.054*	0.038	(1.03–1.14)	1.113*	0.052	(1.01–1.23)
Smoking (reference: no)	4.357**	0.520	(1.571–12.08)	3.198	0.707	(0.80–12.78)
At risk of malnutrition (reference: normal)^a^	5.817***	0.489	(2.23–15.17)	12.405***	0.678	(3.29–46.81)
Physical inactivity (reference: sufficient)^b^	29.101***	1.036	(3.82–221.75)	89.445***	1.390	(5.87–1363.93)

Abbreviations: CI, confidence interval; OR, odds ratio; SE, standard error.

^a^ Assessed by the Short‐Form Mini Nutritional Assessment with a cutoff of 11 points or lower indicating being at risk of malnutrition.

^b^ Measured by the International Physical Activity Questionnaire‐Short Form with a cutoff of < 600 metabolic equivalents of task‐min/week.

* *p* < 0.05.

** *p* < 0.01.

*** *p* < 0.001.

## 4 Discussion

In this study, we aimed to investigate the prevalence of cognitive frailty, as well as the risk factors for cognitive frailty among patients with CKD receiving hemodialysis. The main results were that (1) the prevalences of cognitive impairment, physical frailty, depressive symptoms, and cognitive frailty were 46.8%, 10%, 52.3%, and 9%, respectively; (2) being of a younger age, being female, having undergone hemodialysis for a longer period, engaging in insufficient physical activity, and being at risk of malnutrition were factors associated with cognitive frailty; and (3) the nutritional status and physical activity were the most critical factors that significantly increased the odds of subjects having cognitive frailty.

We reported a 9% prevalence of cognitive frailty in this study cohort. Of these subjects, 46.8% were identified as having cognitive impairment and 10% were physically frail. These results are similar to a systematic review that summarized CKD‐related physical frailty and cognitive impairment in which the prevalence of frailty ranged 7%–14%, and a 14% prevalence of frailty was found in a large hemodialysis cohort (Kutner et al. 2014; Shen et al. 2017). Cognitive functioning declines and depressive symptoms were highly prevalent and were associated with physical frailty in patients with CKD (Greinert et al. 2022; Shen et al. 2017). In addition, the interaction between cognitive impairment and frailty was found among prevalent hemodialysis recipients as well (Anderson et al. 2023). Our results are in line with the existing literature, highlighting the significance of investigating cognitive frailty as a combined concept among patients with CKD receiving hemodialysis. Multidimensional interventions targeting alleviation of cognitive frailty are needed, and integrating strategies of improving physical frailty, cognitive functioning, and depressive symptoms needs to be considered.

This study showed that the patient group with a mean age of 45.9 years on hemodialysis were at lower risk of experiencing cognitive frailty than were their younger counterparts (with a mean age of 39.4 years), while both were at a middle mean age. The result was contrary to the body of knowledge regarding the impact of age on cognitive performance. Most patients with cognitive frailty were relatively young. However, age is a risk factor for cognitive functioning decline, physical frailty, as well as depressive symptoms (Collard et al. 2012; Kelaiditi et al. 2013; Shen et al. 2017). Therefore, based on our results, we were unable to conclude on the relationship between age and cognitive frailty. To examine the risk factor of cognitive frailty, age was adjusted in the multivariate logistic model. Females were found to be more likely to become vulnerable to developing cognitive frailty owing to significantly greater aging‐related loss of lean body mass and hand grip strength compared to men (Suetta et al. 2019). A higher risk of comorbidities associated with CKD may also lead to increased frailty in female patients on hemodialysis. Although a previous study documented no significant differences in sex on hemodialysis practices and treatment regimens, compared to male patients, female patients had significantly higher dialysis rates in the age group of less than 69 years old (Weigert et al. 2020). There was also a significantly higher serum phosphorus level in females than in males, which was associated with higher risks of vascular calcification and cardiovascular diseases (O'Seaghdha et al. 2011). A higher risk of experiencing cognitive frailty should be carefully considered by hemodialysis centers when carrying out hemodialysis on female patients of certain ages. Non‐pharmacological interventions, such as resistance exercises to improve lower‐limb muscle strength (Nascimento Alves et al. 2022) and frailty‐preventative strategies are therefore required to help female patients on hemodialysis preserve their physical strength and adopt a physically active lifestyle (Maia Neves Menezes and Lopes Pereira 2022). Not surprisingly, patients who had received hemodialysis for longer periods were also identified as being associated with cognitive frailty.

After adjusting for age, sex, and years of receiving hemodialysis in our analysis, two significant risk factors were identified: being at risk of malnutrition and being physically inactive. Undergoing hemodialysis adds additional stressors to patients' physiological complexity and performance and leads to an unhealthy lifestyle, such as fluid and diet restrictions and reduced mobility (Ahmad and Al Nazly 2015; Arai et al. 2014). A low‐protein diet is commonly recommended for those with CKD, as renal dysfunction is associated with impaired acid excretion, which puts them at higher risk for experiencing malnutrition (Zha and Qian 2017). A population study (*n* = 2213) that investigated patients with kidney failure in China found that prevalences of sarcopenia, slow walking, and low grip strength were 35.5%, 66.9%, and 66%, respectively (Song et al. 2022). Thus, CKD‐related diet restrictions may play a vital role in patients' frailty syndrome, and special attention should be addressed to prevent an impaired nutritional status.

As to recommendations of the WHO (of ≥ 600 METs‐min/week) regarding sufficient physical activity, we found that patients who were categorized as being physically inactive had a high risk of developing cognitive frailty. Sufficient physical activity by doing at least 150 min/week of brisk walking or 75 min of running is achievable and realistic for patients with CKD receiving hemodialysis, and 55.5% of patients achieved sufficient physical activity in our study cohort. Remaining physically active is important for patients undergoing hemodialysis. However, some barriers and challenges to exercise were identified including comorbid conditions, environmental factors such as safety and weather, costs, time constraints, and a lack of family, peer, and healthcare professional support (Clarke et al. 2015; Kendrick et al. 2019). To motivate patients to exercise, several strategies and suggestions were proposed according to qualitative findings in the literature, for example, providing peer and social support that is linked to enjoyment and social interactions during exercise or organizing group exercises for patients. It is also important to increase patient engagement through informational support to deliver desired individualized information to patients, which would allow them to self‐manage their condition through exercise (Clarke et al. 2015; Kendrick et al. 2019). In addition, studies on intradialytic exercise confirmed its significant positive effect on physical and mental domains of the quality of life (Salhab et al. 2019). Recommendations on precautions of intradialytic exercise in hemodialysis patients were also noted which suggested performing intradialytic exercise within the first 2 h of dialysis to reduce the risk of hypotension. The American College of Sports Medicine also advised avoiding participation immediately following hemodialysis treatment due to an increased risk of hypotension. Exercise on non‐dialysis days was recommended, as was avoiding post‐dialysis exercise (Lambert et al. 2022). Following safety guidance and supervision, intradialytic exercise can be a potential intervention to increase physical activity among patients with CKD receiving hemodialysis.

Our study had several limitations. We recommend caution when ascribing causal relationships of the associated factors with cognitive frailty from this cross‐sectional study. First, the study had a low proportion of older patients (> 65 years, 11.8%), and therefore age‐related functional declines could not be investigated. A young cohort was recruited in this study (mean = 45.3 years) due to the research site being a hospital‐based hemodialysis center, and patients were enlisted through convenience sampling. The prevalence of dialysis in Taiwan is the highest (3593 per million population) in the globe according to the United States Renal Data System (USRDS). There are nearly 430 dialysis centers to respond to the huge demand of dialysis in Taiwan. Most dialysis centers provide transport services (back and forth) for older adults over 65 years old, alongside the long‐term care policy in Taiwan to support older patients receiving hemodialysis. Therefore, the patients recruited from a single hospital‐based hemodialysis center in this study were relatively younger. Although age was appropriately considered to be a confounder in the statistical analyses, there might have been inaccuracies in the magnitude of its true association with the risk of developing cognitive frailty, particularly in older patients (aged > 65 years). Second, unmeasured characteristics and information (e.g., occupation and medication) may have confounded associations of the nutritional status and physical activity with the risk of cognitive frailty. The magnitude of its true association may have been diminished by the effects of residual confounding. It may be challenging to generalize because estimates may have been uncertain, and the wide CIs for estimated ORs. Therefore, additional research with a larger sample size is necessary. Third, adopting a cross‐sectional study design is inadequate for examining this hypothesis on causal effects, such as cognitive frailty leading to physical inactivity and malnutrition or vice versa. Future studies adopting a longitudinal design are warranted to test the causality between these variables. Lastly, there is a potential limitation of recall bias in the study, particularly when participants are asked to remember and report their activities over an extended period.

## 5 Conclusion

Our study identified the prevalences of cognitive frailty alongside cognitive impairment, physical frailty, and depressive symptoms, as well as the risk of malnutrition and physical inactivity being the most critical risk factors associated with cognitive frailty. These results can inform the development of future nursing interventions to tackle the complex condition of cognitive frailty. Strategies to improve physical activity and the nutritional status are warranted and are likely to prevent cognitive frailty among patients with CKD receiving hemodialysis.

## 6 Relevance for Clinical Practice

Healthcare professionals, including nurses, should prioritize interventions that enhance nutritional status and promote physical activity among hemodialysis patients with CKD to mitigate cognitive frailty. Nutritional guidelines should aim to prevent malnutrition and encourage a balanced diet for the CKD population, thereby reducing the risk of cognitive frailty. Additionally, encouraging patients with CKD to engage in sufficient physical activity is crucial. Healthcare professionals and researchers should develop and provide evidence‐based physical activity interventions that address barriers such as environmental factors, time constraints, and lack of support. By doing so, they can help lower the risk of cognitive frailty in this vulnerable population.

## Author Contributions

**Mu‐Hsing Ho:** conceptualization, methodology, formal analysis, writing – original draft. **Pei‐Chi Chen:** conceptualization, data curation, investigation, writing – original draft. **Megan F. Liu:** conceptualization, investigation, writing – original draft. **Jung Jae Lee:** conceptualization, validation, writing – original draft, writing – reviewing and editing.

## Ethics Statement

This study was approved by the institutional review board of Cardinal‐Tien Hospital in Taiwan (approval no. 108‐3‐5‐056; approval date: December 24, 2020).

## Conflicts of Interest

The authors declare no conflicts of interest.

## Data Availability

The data that support the findings of this study are available on request from the corresponding author. The data are not publicly available due to privacy or ethical restrictions.
